# The Relation of the Brain-Derived Neurotrophic Factor with MicroRNAs in Neurodegenerative Diseases and Ischemic Stroke

**DOI:** 10.1007/s12035-020-02101-2

**Published:** 2020-09-17

**Authors:** Ceren Eyileten, Lucia Sharif, Zofia Wicik, Daniel Jakubik, Joanna Jarosz-Popek, Aleksandra Soplinska, Marek Postula, Anna Czlonkowska, Agnieszka Kaplon-Cieslicka, Dagmara Mirowska-Guzel

**Affiliations:** 1 Department of Experimental and Clinical Pharmacology, Medical University of Warsaw, Center for Preclinical Research and Technology CEPT, Banacha 1B Str., 02-097 Warsaw, Poland; 2 Centro de Matemática, Computação e Cognição, Universidade Federal do ABC, São Paulo, Brazil; 3 2nd Department of Neurology, Institute of Psychiatry and Neurology, 02-957 Warsaw, Poland; 4 1st Chair and Department of Cardiology, Medical University of Warsaw, Warsaw, Poland

**Keywords:** Peripheral biomarker, Stroke, miRNA, Neurodegenerative disease, BDNF, Novel treatment, Neurotrophic factor, Neurotrophin, Neurovascular disease

## Abstract

Brain-derived neurotrophic factor (BDNF) is a member of the neurotrophin family of growth factors that plays a crucial role in the development of the nervous system while supporting the survival of existing neurons and instigating neurogenesis. Altered levels of BDNF, both in the circulation and in the central nervous system (CNS), have been reported to be involved in the pathogenesis of neurodegenerative diseases, including Alzheimer’s disease (AD), Parkinson’s disease (PD), amyotrophic lateral sclerosis (ALS), Huntington’s disease (HD), multiple sclerosis (MS), and ischemic stroke. MicroRNAs (miRNAs) are a class of non-coding RNAs found in body fluids such as peripheral blood and cerebrospinal fluid. Several different miRNAs, and their target genes, are recognized to be involved in the pathophysiology of neurodegenerative and neurovascular diseases. Thus, they present as promising biomarkers and a novel treatment approach for CNS disorders. Currently, limited studies provide viable evidence of miRNA-mediated post-transcriptional regulation of BDNF. The aim of this review is to provide a comprehensive assessment of the current knowledge regarding the potential diagnostic and prognostic values of miRNAs affecting BDNF expression and its role as a CNS disorders and neurovascular disease biomarker. Moreover, a novel therapeutic approach in neurodegenerative diseases and ischemic stroke targeting miRNAs associated with BDNF will be discussed.

## Introduction

Along with the nerve growth factor and neurotrophins (NT), brain-derived neurotrophic factor (BDNF) is a member of the NT family that has a crucial role in the development and maintenance of the nervous system. It regulates neurotransmission, neuronal regeneration and morphology, and the functional synaptic plasticity in neurons both in the peripheral nervous system and central nervous system (CNS) by binding tropomyosin receptor kinase B (TrkB) [1, 2]. BDNF has previously been discovered in endothelial cells, muscle tissues, liver, and adipose tissues. Later on, it was also found in immune cells, such as lymphocytes and monocytes [1, 3]. It was stated that the three areas where BDNF is most active are the hippocampus, cortex, and basal forebrain [4, 5]. Altered levels of BDNF, both in the circulating blood and in the CNS tissues, have been reported to be involved in the pathogenesis of neurodegenerative diseases, including Alzheimer’s disease (AD), Parkinson’s disease (PD), amyotrophic lateral sclerosis (ALS), Huntington’s disease (HD), and multiple sclerosis (MS) as well as in ischemic stroke [5–10].

MicroRNAs (miRNAs) are small non-coding RNAs that contain around 22 nucleotides. MiRNAs play a crucial role in diverse biological and pathological processes in cardiovascular disease, diabetes, and neurological disorders and neurovascular disease. They are promising novel disease biomarkers and treatment approaches in cardiovascular diseases and diabetes as well as in neurodegenerative disease and ischemic stroke as they regulate gene expressions [11–17].

In this review, we present a comprehensive overview of the current knowledge of the association between BDNF and miRNAs as well as diagnostic/prognostic/therapeutic value of miRNAs in a range of CNS disorders—including AD, PD, HD, ALS, MS, and ischemic stroke.

## BDNF and MicroRNAs in Neurodegenerative Disease

### Alzheimer’s Disease

Alzheimer’s disease (AD) is the most common known cause of dementia and is characterized by massive neuronal loss, cognitive dysfunction, and memory deterioration, starting usually after the age of 65 years [18, 19]. Associated genetic predispositions have been defined, but the majority of cases are still described as idiopathic, with the underlying neuropathology largely unknown. Pathological changes in AD can be listed as accumulation of amyloid β-42 and phosphorylated tau, the formation of neurofibrillary tangles, low-grade inflammation, and reduced cholinergic functions [20]. Despite scientific advances, there is still a lack of viable biomarkers for AD and with an aging population, the numbers of AD-related deaths are inevitably on the rise.

#### BDNF and Alzheimer’s Disease

The expression of BDNF in the central nervous system and its influence by ischemia, stress, or hypoglycemia are currently well established [21]. BDNF is hypothesized to modulate not only neuronal growth and survival but also AD development. It was observed that AD subjects demonstrate discrepancy in upregulated and downregulated BDNF levels when compared to healthy individuals [22–24]. Additionally, cognitive decline in AD has been associated with BDNF as a result of its diverse biological effects among a wide variety of receptors [25]. A meta-analysis has confirmed a connection between BDNF and AD; however, it also shed light on the implications associated with detection, only being possible in late stages of the disease [26].

#### BDNF Associated MicroRNAs in Alzheimer’s Disease

MicroRNAs are considered by many as a class of gene regulatory elements with roles in the development of AD. They have shown to be essential in the molecular control of neurological development and aging while being associated with AD. MiRNAs have demonstrated an association with AD-related proteins in the brain while remaining at constant levels in both blood and cerebrospinal fluid (CSF) [27].

Croce et al. examined the miRNA changes in AD-affected neurons by using *in vitro* models of AD. Neuropeptide Y (NPY)-pre-treated rat cortical neurons were exposed to Aß25–35 and protein expression was measured after 24 and 48 h along with miR-30a-5p (a member of the miR-30a family and a target of *BDNF*). Results showed that an overexpression of miR-34a, miR-30a-5p, and Let-7d downregulated *BDNF* expression while NPY demonstrated a protective mechanism. Therefore, this study suggested that NPY alters BDNF expression by inhibiting miR-30a-5p expression with a mechanism that more likely contributes to the neuroprotective effect of NPY due to Aß exposure *in vitro* [28]. However, previous study showed that loss in NPY mRNA expression was positively associated to BDNF protein concentration and BDNF levels were inversely associated to miR-195, but not to miR-30a, on the prefrontal cortex of schizophrenic individuals from a postmortem cohort [29].

Memory-associated proteins, including cAMP response element-binding protein (CREB) and BDNF, play a crucial role in learning and are associated with miR-132/212 function and/or regulation, as well as miR-132/212 family plays a crucial role in neural function/plasticity and synaptic plasticity [30, 31]. Genetic deficiency of miR-132/212 caused learning deficiency and decreased spatial memory in miR-132 knockout (KO) mice. Importantly, phosphorylated CREB was found upregulated in the hippocampus while BDNF was downregulated in miR-132 KO mice compared to controls. Hence, both phosphorylated CREB and BDNF may be involved in mechanism of memory formation and retention regulation by miR-132/212 in AD [32].

Kim et al. [33] noted that miR-126 had functional relevance in neurotoxicity. Upregulation of miR-126 increased neuronal toxicity, and inhibition of miR-126 showed neuroprotective effect via increasing NGF expressions such as, IGF-1, NGF, and BDNF. Importantly, the study also searched the mechanism and showed that the neuroprotective impact of BDNF was reduced while the expression of its receptor TrkB was blocked by anti-TrkB. This may suggest that miR-126 can affect BDNF/TrkB signaling pathway. MiR-126 is associated with modulation of IGF-1/PI3K/AKT, p38 MAPK, or ERK cascade in a multitude of non-neuronal cells. Additionally, it is well-known that among the growth factors, BDNF also uses the PI3K signaling pathway [34, 35]. Another important finding of the study was that dysfunctional miR-126 might be a crucial element in nervous system pathogenesis via particularly deregulating PI3K/AKT signaling pathway. Thus, miR-126 can provide a crucial bridge between metabolic disturbances and neurotoxicity through the association with BDNF and their common signaling cascades in the pathogenesis of neurodegenerative disease, including PD and AD [33].

The importance of miR-937 and its relation with BDNF was studied with miR-937-modified mesenchymal stem cells (MSCs) in animal models of AD. Overexpression of antisense-miR-937 in MSCs improved the protective effects of MSCs on AD, through increasing Brn-4 levels. Importantly, the study showed that antisense-mediated inhibition of miR-937 significantly enhanced the BDNF levels. Thus, it suggests that miR-937 deficiency in transplanted MSCs can be beneficial in neuronal repair by increasing BDNF levels [36].

The role of miR-322, a rodent homologue of human miR-424, was investigated by Zhang et al. In Tg2576 transgenic mouse, miR-322 was upregulated and correlated negatively with BDNF levels. The study aimed to explain the mechanism of BDNF expression regulation and clarified that BDNF is a target of miR-322, since it can directly conjugate to BDNF 3′-UTR. In addition, functional experiments revealed that miR-322 mimics downregulate the BDNF level. Knocking down miR-322 expression by inhibitors leads to upregulation of BDNF expression both at mRNA and protein level. Moreover, it was shown that increased miR-322 expression induces the TAU phosphorylation via dysregulating the BDNF-TrkB signaling pathway. These findings have indicated a promising role of miR-322 in miRNA-based therapeutic strategies in AD [37].

##### The Importance of miR-206 and Its Link to BDNF in AD

**Fig. 1:**
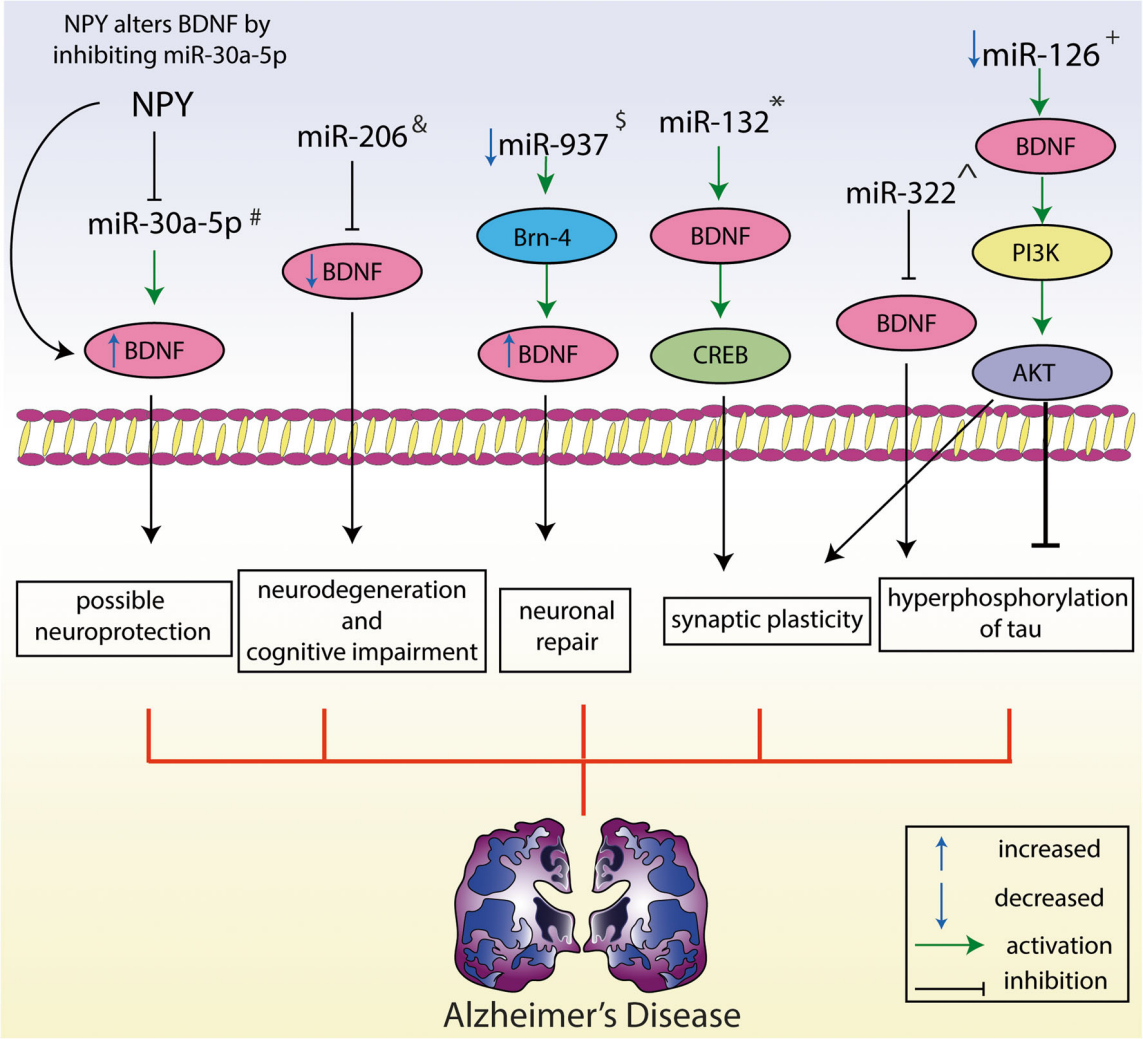
Possible mechanisms of targeted microRNAs involved in BDNF signaling in Alzheimer’s disease. ^#^Based on *in vitro* study [28]; ^&^based on animal model and human study [40, 42]; ^$^based on animal study [36]; *based on animal study [32]; **^**based on animal study [37]; ^+^based on *in vitro* and animal study [33]. Abbreviation: NPY, neuropeptide Y; miR, microRNA; BDNF, brain-derived neurotrophic factor; AChE, acetylcholinesterase; C/EBPa, CCAAT/enhancer-binding protein alpha; CREB, cAMP response element-binding protein; PI3K, phosphoinositide 3-kinase; AKT, protein Kinase B

For the first time, Lee et al. showed that miR-206 modulates BDNF and memory function in AD mice and intracerebral administration of an antagomir miR-206 (an inhibitor of miR-206), in Tg2576 mice. Inhibition of miR-206 showed enhanced BDNF levels and memory performance, including the improvement of hippocampal synaptic density and neurogenesis. Antagomir miR-206 was utilized as a treatment and administered intranasally. Intranasal administration of antagomir miR-206 attained to the brain and enhanced BDNF levels and improved memory impairment in AD animal model. Collectively, these results demonstrated that miR-206 can involve in the pathogenesis of AD by inhibiting BDNF expression. Using antagomir miR-206 increased BDNF levels and can be a promising treatment approach in AD to regulate BDNF expression [38]. The same research group further confirmed their results and showed that olfactory mucosal miR-206 level can be determined, and it can provide as a diagnostic biomarker of early AD, including MCI [39, 40]. Further, Tian et al. also aimed to assess whether miR-206 might alter BDNF in the process of AD pathogenesis. Interestingly, miR-206 expression was higher in the hippocampus, plasma, and CSF of APP/PS1 mice as compared to wild-type (WT) mice, whereas BDNF level was lower in the hippocampal tissue, plasma, and CSF of APP/PS1 mice than in WT mice. The study reported that miR-206 can directly target BDNF by binding 3′UTR. Mimic of miR-206 administration decreased the BDNF levels, whereas the antagomir-206 usage increased both intracellular and secreted BDNF levels [40]. Transgenic APP/PS1 mice with memory impairment showed significantly increased miR-206 levels both in the hippocampus and cortex compared to WT control. Chronic usage of donepezil improved memory function and BDNF dysfunction, while downregulating the miR-206 expression. In turn, miR-206 agonist (agomiR-206-3p) significantly weakened the anti-dementia effects of donepezil. These findings provide that BDNF function is closely related to donepezil treatment and inversely correlated with miR-206 levels [41]. Later, miR-206 was evaluated in order to find a potential prognostic marker of progression of amnestic mild cognitive impairment (aMCI) to AD. The study analyzed the clinical data of 458 patients recruited from MASHB study (mild cognitive impairment and Alzheimer’s disease study in Heibei province) collected twice: at the baseline and at the 5-year follow-up. It was found that miR-206 levels were higher, and BDNF levels were lower in patients with AD conversion. According to multivariate regression analysis, higher concentrations of miR-206 and lower BDNF levels were significantly associated with conversion from aMCI to AD; thus, they may act as an independent prognostic biomarker [42]. MiR-206 belongs to the miR-1 family and recently, many studies analyzed the effect of miR-206 in neurodegenerative disease. It is one of the most well-studied miRNAs in AD. Several reports have shown that miR-206 is involved in the pathophysiology of AD due to suppression of the BDNF expression in the brain. Studies have found that patients with AD and AD animal models have increased miR-206 levels in the brain tissue, which causes memory loss by decreasing both BDNF protein levels and gene expressions in the CNS [39, 40, 42] (Table 1, Fig. 1).

**Table 1:** Evaluating studies targeting microRNAs involved in the BDNF signaling in neurodegenerative diseases and ischemic stroke

Refs	Disease	Study type (*in vivo/in vitro/in silico/*human)	Studied miRNAs	Modulation	Relation with BDNF and conclusion
[28]	Alzheimer’s disease	*In vitro*, using primary rat cortical neurons	miR-34, miR-30, Let-7d	BDNF	Overexpression of miR-34a, miR-30a-5p, and Let-7d was shown to downregulate BDNF expression
[32]	Alzheimer’s disease	miR-132/212 knockout mice	miR-132/212	BDNF, CREB, and MeCP2	Changes in BDNF, CREB, and MeCP2 were identified in the miR-132/212-deficient mice, providing a potential mechanism for promoting memory loss.
[33]	Alzheimer’s disease	Animals and primary cell culture, BDNF-treated cortical neurons	miR-126	IGF-1, NGF, and BDNF	MiR-126 affects the BDNF/TrkB signaling cascade
[38]	Alzheimer’s disease	Tg2576 AD transgenic mice and human AD brain samples	miR-206	BDNF	Antagomir miR-206 prevented the detrimental effects of amyloid-b42 on BDNF
[43]	Alzheimer’s disease	Human: 32 patients with MCI and 48 with dementia AD vs 40 healthy controls Animal: miRNA expression profiling was done on hippocampus species from APP/PS1 mice In silico/in vitro: SH-SY5Y cells	miR-613	BDNF	MiR-613 can directly target BDNF by binding to the 3′-UTR and miR-613 negatively regulates the expression of BDNF *in vitro*
[36]	Alzheimer’s disease	Transplantation of mesenchymal stem cells, AD mice model	miR-937	BDNF, Aβ, Brn-4 protein levels	Depletion of miR-937 in transplanted MSCs increased BDNF levels.
[40]	Alzheimer’s disease	Embryonic APP/PS1 transgenic mice model of AD	miR-206	BDNF	Mimic of miR-206 decreases BDNF levels in a transgenic mouse
[41]	Alzheimer’s disease	APP/PS1 mice	miR-206	BDNF	Donepezil treatment decreased the hippocampal and cortical miR-206-3p expression in APP/PS1 mice, AgomiR-206-3p administration further exacerbated the memory impairments and BDNF dysfunction in APP/PS1 mice
[42]	Alzheimer’s disease	Human study; 458 patients with aMCI were included: aMCI-stable group (*n* = 330) and AD-conversion group (*n* = 128).	miR-206, miR-132	BDNF and SIRT1	Serum miR-206 and its target BDNF were significant independent predictors for AD conversion. Increased serum miR-206 level might be a potential predictor conversion from aMCI to AD.
[44]	Alzheimer’s disease	MiR-29c and BDNF in the CSF of patients with AD and control individuals	miR-29c	BDNF	MiR-29c exerts a neuroprotective effect via the BDNF/TrkB/Erk signaling pathway
[37]	Alzheimer’s disease	AD mouse brain and *in silico* analysis	miR-322	BDNF	MiR-322 can directly conjugate to BDNF 3′-UTR. MiR-322 promotes Tau phosphorylation via negatively controlling BDNF–TrkB receptor activation.
[54]	Parkinson’s disease	*In silico*, *in vitro* model PC12 cell line from rat pheochromocytoma	miR-34a, miR-141, miR-9	BDNF, BCL2, and SIRT1	MiR-34a could be a BDNF-targeting miRNA in PD.
[60]	Parkinson’s disease	PD patients and normal controls and neuroblastoma SH-Y5Y cells	miR-21	BDNF	DHA increases BDNF and promotes expression of PPARa through inhibiting miR-21.
[55]	Parkinson’s disease	SNpc of MPTP-induced PD mice	miR-30e	BDNF	MiR-30e agomir administration attenuated the marked increase of inflammatory cytokines, such as TNF-α, COX-2, iNOS, and restored the decreased secretion of BDNF in SNpc.
[68]	Huntington’s disease	Post-mortem brain tissue samples of unaffected individuals and HD patients	miR-132, miR-124a	REST, BDNF	Increased nuclear REST results directly in decreased levels of miR-132 and miR-124a and indirectly via inhibition of BDNF expression.
[71]	Huntington’s disease	*In silico*	miR-10b-5p	BDNF	Upregulation of miR-10b-5p can have a neuroprotective effect due to its target to BDNF/CREB1 and in response to the mutation in the huntingtin gene
[81]	Amyotrophic lateral sclerosis	SOD1-G93A primary microglia obtained from brain cortex of P0/P1 B6.Cg-Tg(SOD1-G93A)1Gur/J mice	miR-125b	BDNF	MiR-125b inhibition significantly upregulates BDNF
[94]	Multiple sclerosis	Animal model with EAE	miR-155-5p	BDNF	Strong negative correlation between BDNF mRNA and miR-155-5p expression was found. Protective role of IL-17 was mediated by the downregulation of pro-inflammatory miR-155-5p and subsequent upregulation of its target mRNAs BDNF.
[96]	Multiple sclerosis	Human brain samples who died due to MS (*N* = 16)/healthy individuals brain samples (*N* = 5)	miR-191	BDNF	Reduced miR-191 resulted in increased levels of BDNF
[95]	Multiple sclerosis	Animal model with EAE	miR-125a	BDNF	Inverse correlation between miR-125a and BDNF was observed.
[106]	Ischemic stroke	First *in silico*, then validation the results with *in vivo* mouse model of ischemia	Let-7i	Pgrmc1 and BDNF	Let-7i inhibitor (antagomir) restored Pgrmc1 expression and resulted in a marked increase in mature BDNF level.
[108]	Ischemic stroke	*In vitro* human early EPCs, also referred to as CFU-Hill cells treated with BDNF	miRNA profiling: 6 miRNAs were significantly downregulated: miR-4716-5p, miR-3928, miR-433, miR-1294, miR-1539, miR-19b-1*, and 10 miRNAs were significantly upregulated: miR-432, miR-4499, miR-3911, miR-1183, miR-4669, miR-636, miR-4717-3p, miR-4298, miR-485-5p, miR-181c	BDNF	BDNF negatively regulated miR-3928 and positively regulated miR-636 and miR-485-5p. Importantly, BDNF treatment significantly suppressed miR-433 and promoted miR-181c levels
[110]	Ischemic stroke	Circulating miRNAs profiles examination in the ischemic stroke patients, *in silico* analysis and validation of the results with focal cerebral ischemia in mice	miRNA profiling results: 24 miRNAs were significantly upregulated or downregulated hsa-let-7a*, let-7f-2*, miR-1254, miR-1468, miR-15a*, miR-192*, miR-224*, miR-29a*, miR-223, miR-324-5p, miR-891b, miR-142-3p, miR-143, miR-144, miR-190, miR-192, miR-19b, miR-210, miR-215, miR-29b, miR-342-3p, miR-574-3p, miR-589, miR-720; After IPA analysis: miR-210, miR-589, miR-891, miR-223, miR-15, miR-143, miR-574, miR-192*, miR-1254, and let-7* were the highest score. Further miR-210 was studied in the mouse model.	BDNF	Delivery of lentivirus-mediated miR-210 to the ischemic brains of mice and upregulated mature/ BDNF/pro-BDNF ratio.
[111]	Acute ischemic stroke	miR-124 in regulating BDNF was determined in acute ischemic stroke patients and controls, *in silico* prediction, further *in vitro* confirmation	miR-124	BDNF	Both miR-124 and BDNF can be a promising novel biomarker of ischemic stroke, miR-124 and BDNF could be predictors of stroke severity and inhibitors of miR-124 could be used as a potential approach for increasing BDNF serum levels.

*BCL2*, B-cell lymphoma 2; *SIRT1*, Sirtuin 1; *REST*, RE1-silencing transcription factor; *CREB*, cAMP response element-binding protein; *MeCP2*, methyl CpG binding protein 2; *Abeta*, amyloid beta peptide-Aβ; *IGF-1*, insulin-like growth factor 1; *NGF*, nerve growth factor; *BDNF*, brain-derived neurotrophic factor; *miRNA-miR*, microRNA; *EPCs*, endothelial progenitor cells; *CFU-Hill*, colony-forming units-Hills; *Pgrmc*, progesterone receptor membrane components; *EAE*, experimental autoimmune encephalomyelitis; *MS*, multiple sclerosis; *IL-17*, interleukin 17; *TNF-α*, tumor necrosis factor alpha; *COX-2*, cyclooxygenase-2; *iNOS*, inducible nitric oxide synthase; *SNpc*, substantia nigra pars compacta; *MPTP*, 1-methyl-4-phenyl-1,2,3,6-tetrahydropyridine; *PPAR*, peroxisome proliferator-activated receptor; *TrkB*, tropomyosin receptor kinase B; *aMCI*, amnestic mild cognitive impairment; *AD*, Alzheimer’s disease; *ERK*, extracellular signal-regulated kinase; *IPA*, ingenuity pathway analysis

In a human study, 32 patients suffering from mild cognitive impairment (MCI) and 48 with dementia of Alzheimer’s disease type (DAT) were compared to 40 healthy individuals. Both MCI and DAT patients had a lower number of mRNA BDNF transcripts as well as expression of BDNF protein in both plasma and CSF. To determine which miRNA may regulate the process of BDNF transcription, the miRNA expression profiling was done on hippocampus species from APP/PS1 mice. Being never reported before miR-613 was chosen based on *in silico* prediction for further study. MiR-613 levels were significantly higher in MCI and DAT patients. The study showed that miR-613 can target *BDNF* gene by binding the 3′-UTR. Additionally, the negative modulation of miR-613 on BDNF was confirmed, as miR-613 significantly decreased the both BDNF mRNA and protein production *in vitro* [43]. Yang et al. [44] investigated and compared concentrations of miR-29c and BDNF in the CSF of patients with AD and control individuals. The study revealed that along with decreased miR-29c concentrations, BDNF protein expression was also downregulated. Moreover, the authors reported a higher expression of DNA methyltransferase 3 (DNMT3), which is a target of miR-29c in the CSF of AD patients. *In vitro* experiments suggested that possible epigenetic mechanisms underlying AD have at least partial origin in dysregulation of DNMT3 expression caused by miR-29c at the transcriptional level by targeting its 3′UTR. Consequently, increased BDNF promoter methylation by its inhibitor DNMT3 possibly leads to downregulated protein expression. MiR-29c has an important role in neuronal growth by targeting the TrkB/Erk signaling pathway, since upregulation of miR-29c results in increased expression of TrkB and Erk proteins. It is important to note that BDNF/TrkB signaling is weakened in AD, and BDNF and TrkB have a crucial role in the CNS by supplying trophic support to nerves [45]. Thus, this may suggest that miR-29c utilizes a neuroprotective effect via the BDNF/TrkB/Erk signaling pathway and it can be a possible therapeutic approach for the management of AD [44]. As abovementioned, miR-206 was studied in patients from MASHB study and higher miR-206 levels and lower BDNF levels were significantly associated with conversion from aMCI to AD [46].

MiR-206 is a promising biomarker which has been intensively studied in AD. Both animal and human studies have shown its importance in AD targeted with BDNF. Consequently, in animal studies, antagomiR-206 was used as a treatment and it further ameliorated the memory loss and BDNF deficiency in mice. Taken together, published data may suggest that the miR-206 inhibitor can be a potential novel therapeutic approach for AD and should be further analyzed.

### Parkinson’s Disease

Parkinson’s disease (PD) is an idiopathic neurodegenerative disorder that currently affects more than two million people worldwide and second-most common neurodegenerative disorder after AD. It is characterized by progressive degeneration of nigrostriatal dopaminergic neurons and the development of intracellular proteinaceous aggregates, which impairs by both motor functions (tremor, rigidity, and bradykinesia) and non-motor functions (cognitive decline) [47].

#### BDNF and Parkinson’s Disease

Numerous pre-clinical and clinical studies have shown alterations of BDNF levels among the PD individuals, showing its potential role in the pathogenesis of the disorder. BDNF is a critical factor for vitality and maturation of dopaminergic neurons which loss in substantia nigra pars compacta results in a dopaminergic deficiency in the striatum. The protective effect of BDNF supports neuronal development and survival of nigrostriatal neurons [48]. It inhibits nigrostriatal apoptosis via BDNF/TrkB signaling and is essential for the maturation and functioning of fully developed dopaminergic neurons [1]. Overexpressed neuronal protein alpha-synuclein—a main component of Lewy bodies—downregulates the BDNF expression level [47]. Moreover, among patients with PD, the BDNF concentrations were found to be decreased in brain tissue of AD postmortem and in the substantia nigra of PD patients [49]. Additionally, BDNF levels were significantly lower in the newly diagnosed patients with PD. Not only pharmacological treatment with Levodopa results in an increased level of BDNF but also alternative non-pharmacological interventions including cognitive rehabilitation speech therapy and physiotherapy may potentially have a positive effect on the BDNF concentration. Moreover, it was demonstrated in rat model that *BDNF* gene therapy of nigral dopamine neurons prevents degeneration of striatum at the early stage of PD which underlines the potential therapeutic role of BDNF [50, 51].

#### BDNF Associated MicroRNAs in Parkinson’s Disease

MicroRNAs have been associated with multiple pathways of PD pathophysiology. MiR-939 and miR-26a have shown to be involved with oxidative stress affecting the pathogenesis of PD-related neuroinflammation [52]. Leucine-rich repeat kinase 2 (LRRK2) is an important factor involved in the etiology of PD, *LRRK2* mutations are the major reason of inherited and sporadic PD, and overexpression of miR-205 showed an association with the abnormal upregulation of LRRK2 in PD brains [53]. Importantly, BDNF is known as a key protector factor against neurotoxin-induced neurodegeneration of dopaminergic neurons and cell apoptosis [49]. A recent study confirmed such observation and also aimed to analyze many different miRNAs and related target genes by using 1-methyl-4-phenylpyridinium (MPP^+^) model, a dopaminergic neurotoxin which causes parkinsonian-like symptoms. The study used *in vitro* model of PD using a PC12 cell line from rat pheochromocytoma. MPP+ caused the loss of cell viability and enhanced apoptosis and reactive oxygen species (ROS) overexpression. The research showed downregulation of *BCL2*, *BDNF*, and *SIRT1* due to MPP+ exposure. Importantly, miR-34a, miR-141, and miR-9 were increased following MPP+ toxicity. For the first time, this study demonstrated that miR-34a could be a BDNF-targeting miRNA in humans by using *in silico* approach. The authors suggested that the upregulation of miR-34a, miR-141, and miR-9 can target these genes (*BCL2*, *BDNF*, and *SIRT1*) and affect their expressions [54].

Downregulation of miR-30e was found in substantia nigra pars compacta (SNpc) of MPTP-induced PD animal model. Agomir of miR-30e administration significantly improved motor function and improved the dopaminergic neurons depletion, specifically by targeting and decreasing the inflammasome activity of protein-coding gene, *NLRP*3 and increasing the inflammatory cytokines including COX-2, TNF-α, and iNOS. It suggests that miR-30e can be neuroprotective in MPTP-induced PD animal model. Importantly, the study showed that miR-30e agomir treatment improved the decreased BDNF production in SNpc [55]. Another study showed that melatonin can increase miR-30e expression and upregulation of miR-30e can significantly inhibit the NLRP3 inflammasome pathway whereas the positive effect of melatonin was neutralized by miR-30e inhibitor. Thus, it showed association between melatonin and the miR-30e/NLRP3 inflammasome signaling cascade [56]. It is well known that anorexigenic proopiomelanocortin (POMC) neurons function to the hypothalamic paraventricular nucleus (PVN) and ventromedial nucleus of the hypothalamus (VMN), where alpha-melanocyte-stimulating hormone (α-MSH) is produced, by activating the melanocortin-4 receptor (MC4R). Besides, the connection between BDNF and MC4R signaling was documented with several studies [1]. MC4R has a crucial role in regulating energy metabolism, and obesity has been observed in mice with mutations in the gene for *BDNF* [57]. BDNF is expressed at high levels in the VMN, where its expression is modulated by MC4R signaling. Thus, it may suggest that MC4R activation in the VMN can control BDNF expression [57, 58]. Besides, altered miR-30e expression in hypothalamus after a chronic caloric restriction and HFD in animal model was documented [59]. Taken together, it can be hypothesized that BDNF may have a link with miR-30e via MC4R from the hypothalamus and NLRP3 inflammasome signaling pathway. Future studies are needed in order to discover the exact mechanism between BDNF and miR-30e (Table 1).

Another study in PD investigated the intricate mechanism of relation between BDNF and miR-21. Both markers were assessed in isolated peripheral blood mononuclear cells (PBMC) from patients and control individuals as well as in SH-SY5Y cells (an *in vitro* model for PD). Decreased expressions of peroxisome proliferator–activated receptor alpha (PPARα—an isotype of PPAR, which is expressed in the brain, and involved in excitatory neurotransmission) and increased miR-21 levels were found in PD patients compared with normal controls. Further, the study investigates the effect of docosahexaenoic acid (DHA—a fatty acid, which plays a critical role in the preservation of the nervous system and is involved in the treatment of neurodegenerative diseases) and inhibitory effect of miR-21 on PD by using neuroblastoma SH-Y5Y cells. DHA affected PPARα by activating RXRα and promoting expression of PPARα through inhibiting miR-21. Importantly, the study showed that DHA increased neurotrophic factors such as GDNF and BDNF. Thus, authors hypothesized that DHA upregulates PPARα expression by miR-21 inhibitor and activates RXRα, which reveals induced BDNF, functioned as a neuroprotective factor [60].

To date, there’s a limited number of studies assessing the relation between miRNA and BDNF in PD. Essentially, only miR-34a, miR-141, miR-9, miR-21, and miR-30e were analyzed based on BDNF modulation. Among these miRNAs, miR-34a was found as a direct target of BDNF throughout *in silico* analysis. Further, this prediction was confirmed throughout an experimental analysis. Moreover, miR-34a was suggested as an indicator of the PD-related mechanisms, including oxidative stress and apoptosis. Thus, further studies should confirm these important results.

### Huntington’s Disease

Huntington’s disease (HD) is a progressive neurodegenerative disorder which is inherited in an autosomal dominant genetic condition. Ultimately, HD is caused by an expanded CAG repeat in the huntingtin gene (*HTT*) which results in abnormal protein processing and aggregation [61]. Clinically, HD presents with distinctive chorea, dystonia, incoordination, cognitive decline, and psychiatric behaviors [61].

#### BDNF and Huntington’s Disease

The role of BDNF has been extensively investigated since the striatum does not produce BDNF, as the main source of BDNF is the cortex or thalamus and mesencephalon, and it depends on it for proper functioning [6, 61, 62]. It is hypothesized that BDNF transfer from the cortex to the striatum is attenuated in an animal model of HD. Therefore, the reduction in cortical BDNF delivery would result in diminished activity of the cortex and striatum synaptic activity and resulting in synaptic loss. Additionally, another animal study presented increased vulnerability of these particular neurons as a result of the altered release of BDNF-containing vesicles in the brain. In light of the aforementioned, BDNF was tested on HD mouse models. Results displayed an improvement in disease phenotypes [63]. Moreover, molecular findings have demonstrated the link between *HTT* and RE1-silencing transcription factor *(REST*), a transcriptional repressor, which plays a key role in neuronal differentiation and CNS development. WT HTT protein can activate BDNF and sequester REST/NRSF protein in the cytoplasm. However, the presence of mutant *HTT* (*mutHTT*) results in reduced interaction between *mutHTT* and *REST* leading to a redistribution of *REST* into the nucleus. Of which, this shows the mutation of huntingtin causes a loss of transcription of neuronal genes, such as *REST* [64, 65]. The pathophysiology of HD is still unclear, and there is no treatment for it currently; thus, further studies are needed to discover neuroprotective therapy for HD.

#### BDNF Associated MicroRNAs in Huntington’s Disease

MiRNAs have shown to be abnormal in various regions of the brains of HD patients [66]. Previous study used the post-mortem brains of HD patients to show that miR-22 has protective mechanisms against the disease [67]. These abnormalities have corresponded to clinical tests in the prodromal stage of the disease. Because of their proposed function in communication between neuronal and peripheral tissues, circulating miRNAs have specifically shown to be a topic of interest in HD studies. Alterations in post-transcriptional regulation have shown to be a potential mechanism underlying repressed miRNA expression in patients suffering from this disease [66].

Johnson et al. [68] showed that enhanced levels of nuclear REST in HD neurons cause to alterations in the neuronal transcriptome both directly, by inhibition of target gene expression, such as BDNF, and indirectly, by modulation of miRNA expression, such as miR-132 in neurons. The authors compared miRNA expression in post-mortem brain tissue samples of unaffected controls and HD patients, which revealed significant differences in miRNA levels. Known *REST* targets, miR-132, and miR-124a levels were significantly downregulated in the HD samples. Increased nuclear REST resulted directly decreasing the levels of miR-132 and miR-124a and indirectly via inhibition of BDNF expression consequently leading to inhibition of neurite outgrowth [69]. The same group hypothesized that REST can target BDNF, miR-124, and miR-132. BDNF is a well-known neuronal survival promoter, while miR-124 and miR-132 post-transcriptionally can inhibit non-neuronal genes or genes involved in blockage of neuronal function. BDNF can positively regulate miR-124 but not miR-132 [70]. Finally, in 2014, *in silico* results showed the important role of miR-10b-5p and its target gene *BDNF* in HD. The study re-analyzed the previously published next-generation sequencing data in array express. Bioinformatic analysis found that BDNF is in the centrum of the miRNA-mRNA regulatory network and might be post-transcriptionally functioned by increased miR-10b-5p and miR-30a-5p expressions. Besides, the upregulation of miR-10b-5p in HD can have a neuroprotective effect due to its target to *BDNF*/*CREB1* and in response to the *mutHTT* [71] (Table 1). A limited number of studies have analyzed the relation of BDNF with miRNAs in HD. So far, only miR-22, miR-132, and miR-124 have been determined in experimental analyses, whereas miR-10b-5p was found in bioinformatic analysis which should be confirmed in the future in experimental model.

### Amyotrophic Lateral Sclerosis

Amyotrophic lateral sclerosis (ALS) is a fatal adult-onset disease affecting both the upper and lower motor neurons causing their degeneration due to reduced delivery of major trophic molecules, which are crucial regulators of neuronal survival and regeneration. ALS most frequently affects individuals sporadically (90% of cases); however, less commonly, patients are targeted in an autosomal dominant, inherited fashion. Ultimately, death ensues around 3–5 years after diagnosis and currently, the disease is incurable [72].

#### BDNF and Amyotrophic Lateral Sclerosis

BDNF as a member of the neurotrophin family belongs to the main growth factors binding to the TrkB receptor, supporting regeneration and survival which was evaluated both in *in vitro* and *in vivo* models of injured neurons [73, 74]. However, most trials aiming to assess the differences in BDNF levels among ALS patients failed to confirm such results. Based on a study conducted on spinal cord tissue obtained from SOD1 (G93A) mice (serving as a murine model of ALS), levels of BDNF showed a significant reduction compared to control mice [75]. Recent studies have confirmed the crucial role of BDNF in promoting motoneurons survival; therefore, BDNF as a potential treatment for ALS is a highly studied area of research. *In vitro* studies of BDNF supplementation have shown to ameliorate neurodegenerative changes of ALS by inhibiting apoptosis and restoring calcium homeostasis. Animal models of ALS treated with BDNF showed significant survival of both spinal motor neurons and corticospinal neurons [76]. A phase 1 clinical trial showed positive delivery of BDNF by intrathecal administration; however, high doses also had notable behavioral implications but further clinical studies have failed to produce more than negligible results on halting disease progression [77].

#### BDNF Associated MicroRNAs in Amyotrophic Lateral Sclerosis

MiRNA dysregulation has been described in ALS pathology. Numerous ALS-associated genes have found to be associated with miRNA processing [78]. Mutations in *TDP-43*, *FUS*, and *SOD1* stimulate cellular stress resulting in reduced miRNA expression and neurodegenerative process. A study using pluripotent stem cells further presented 15 downregulated miRNAs including miR-34a and miR-504 [79]. More recently, a study on muscle tissue from ALS patients showed upregulated levels of miR-132 and miR-125b [80]. Studies showed that TDP-43 and *FUS* can function miR-132 and NF-kB pathway activation in microglia can modulate miR-125b in ALS [79–81]. Thus, miRNAs could be a potential strategy in the early diagnosis and treatment of ALS. On the other hand, limited studies investigate the relation between BDNF and miRNAs in ALS. Microglia are involved in ALS toxicity and neuronal protection. Parisi et al. described the M1/M2 functional imprinting of primary microglia and the importance of P2X7 and miR-125b in ALS microglia activation in their second publication. They showed that upregulated miR-125b led to an uncontrolled toxic M1 microglial reaction [82]. Extracellular ATP is involved in inflammation by activation of P2 receptors, including P2X7 receptors. Activation of P2X7 by its agonist 2′-3′-*O*-(benzoyl-benzoyl) ATP (BzATP or ATP) in microglia can indicate morphological transformation and elevation of proinflammatory cytokine expression as well as induction of cell apoptosis [83]. Parisi et al. showed that BzATP fails in inducing BDNF mRNA expression in primary microglia; besides, inhibitor of miR-125b administration significantly upregulated BDNF, with no direct dependence of BzATP. It suggests that the neuroprotective effect of miR-125b inhibition can be due to both downregulation of proinflammatory mediators and the stimulation of M2-activator parameters, such as BDNF in ALS [81]. The expression levels of miR-1, miR-133a, miR-155, miR-206, and miR-378 for their role in the pathophysiological processes in ALS patients after the lineage-negative (Lin-) cells administration were investigated. Lin- cells’ administration increased expression of miR-206 and decreased miR-378 both in the CSF and plasma. On the other hand, no significant differences in the levels of NT (NGF and BDNF) growth factors were found neither before nor after Lin- cells transplantation. The study did not discuss the potential correlation between BDNF and alteration of miRNAs [84] (Table 1). To date, only one study with miR-125b showed the modulation of BDNF signaling of miRNAs in ALS. As the disease is incurable and death inevitable 3–5 years after the diagnosis, more extensive investigations are required in order to discover potential novel early biomarkers and therapeutics.

### Multiple Sclerosis

Multiple sclerosis (MS) is a progressive, chronic, autoimmune inflammatory and degenerative process that damages CSN. MS is characteristic for patients with the presence of plaques and lesions in the brain and spinal cord, primarily the white matter of the brain [3, 7, 85, 86]. The mechanism of MS development remains poorly understood.

#### BDNF and Multiple Sclerosis

A previous study utilized bone marrow chimeras and suggested that CNS resident cells are the main source of BDNF, which plays a neuroprotective role against autoimmune demyelination [87]. However, it was suggested that the immune system provided both destructive and protective factors in MS and BDNF. Hence, the process may potentially act as an immune-mediated defense of neurons in MS lesions [88]. A study by Stadelman et al. demonstrated that in MS lesions, BDNF is initially presented in T cells and macrophages, confirming the capability of the human immune system in producing BDNF. As higher levels of cytokines are a prominent feature in MS, they tend to negatively affect circulating BDNF levels [89]. Low BDNF levels tend to correlate with high IL-6 levels and worse scores in cognitive tasks among MS individuals indicating [90]. Collectively, both animal and human studies have displayed the ability and importance of BDNF to regulate CNS myelination in MS [91, 92].

#### BDNF Associated MicroRNAs in Multiple Sclerosis

Despite being studied for years, the cause of MS is still unknown. Studies showed that miRNAs are involved in physiological and pathological processes in MS [93]. Over the past decade, many studies investigated miRNAs in MS patients. An overwhelming 500 miRNAs have been reported as dysregulated in MS. However, limited studies investigate the relationship of BDNF with miRNAs in MS [93].

In animal MS model, experimental autoimmune encephalomyelitis (EAE), the role of miR-155-5p in the pathogenesis of MS was shown. IL-17 cytokine microinjection to mice brain showed protective activity by indirectly decreasing miR-155-5p expression in animal model of MS. Importantly, a strong negative correlation between BDNF mRNA and miR-155-5p expression levels was found [94]. Another study performed miRNA expression profiling on isolated splenocytes from 6 EAE mice compared to 6 controls. The microarray results showed that 9 miRNAs had different expressions between the groups. Two target genes of miR-125a and miR-99b were selected for further analysis—BDNF and leukemia inhibitory factor (LIF). Interestingly, an inverse correlation between miR-125a and BDNF and between LIF and miR-99b was observed [95].

The miRNA profiling was performed in brain specimens from 16 MS patients and 5 healthy donors. 15 miRNAs were significantly altered in MS patients and 14 different pathways were predicted to be altered due to differentially expressed miRNAs in MS patients (with the strongest correlation with MAPK pathway). Since miR-191 was the most significantly decreased miRNA in MS patients, target prediction was used to determine the potential targets of the miRNA. *WSB1* gene (connected with ubiquitin-mediated proteolysis) and SOX4 mRNA (involved in TGF-ß signaling pathway) presented significantly negatively correlated with miR-191. As a summary, the study showed that BDNF mRNA transcripts were significantly reduced in the normal appearing white matter (NAWM) of MS patients and presented the significant inverse correlation with miR-191, which suggested that reduced miR-191 resulted in increased levels of BDNF [96]. Further studies can consider using anti-miR-191 therapy in order to upregulate the BDNF levels since BDNF promotes neuronal growth and survival and has been demonstrated to be a potential therapy for decreasing neurodegeneration in MS [91].

Abovementioned, there are limited studies examining the relationship between BDNF levels and miRNA expression in MS. Looking to MS, miR-191 seemed to be the most attenuated, whereas in EAE mice, levels of miR-125a seemed to be of greater significance. Importantly, both miR-191 and miR-125 regulate oligodendrocyte maturation and myelination. Ultimately, further analysis is essential in order to establish the importance of these miRNAs in relation to BDNF.

## Ischemic Stroke

Stroke is a major cause of death and disability globally and is the second most-common cause of death worldwide [97]. Essentially, strokes result in a dysfunction of the areas of the brain that are deprived of oxygenated blood. During oxygen deficiency, neuronal networks are damaged, and patients suffer from an aphasia, impairment of sensation, later on movement and cognitive impairment [15, 98, 99].

### BDNF and Ischemic Stroke

Recently published studies presented the crucial role of BDNF in ischemia, suggesting its correlation with post-stroke mobility. It was observed that BDNF levels during the first 24 h of stroke were significantly higher among patients under 65 years compared to older individuals. Additionally, low BDNF concentrations were associated with clinical status during 90-day follow-up [100]. It shows that BDNF levels in the acute phase of ischemic stroke may possess a prognostic value for the functional status of the patient [8, 9]. Studies noted that deficiency of BDNF is related to more severe stroke pathophysiology, as BDNF has a crucial role in development of the nervous system as well as promoting neuronal differentiation, cellular survival, and neurogenesis [10, 101, 102].

#### BDNF Associated MicroRNAs in Ischemic Stroke

It is emerging in the literature on miRNAs as significant biomarkers in stroke [15]. Low levels of serum miR-320b have shown to be an important risk factor for carotid atherosclerosis, a prodromic event that commonly leads to stroke [103]. The upregulation of miR-146a has shown a significant link with neuroprotection from cerebral ischemia [104]. The downregulation of miR-30a has shown to reduce ischemic injury by enhancing beclin-1-mediated autophagy [105]. Clinical results discussing the relationship between BDNF and miRNAs in stroke in humans are very limited. Also, limited number of *in vivo*, *in vitro*, and *in silico* analysis showed the relation between miRNAs and BDNF in ischemic stroke.

Nguyen et al. aimed to investigate the importance of Let-7i in modulating the protective effect of progesterone in ischemia, as it is known that progesterone has neuroprotective function in ischemic stroke [106, 107]. Bioinformatic analysis was performed to select the miRNA; based on *in silico* prediction, they analyzed the effect of Let-7i on both progesterone receptor membrane component 1 (Pgrmc1—classical progesterone receptor and a multi-functional protein) and BDNF in animal model. The study showed that Let-7i mimic transfection reduced mRNA levels of both Pgrmc1 and BDNF. On the other hand, in *in vivo* model of ischemia, administration of both progesterone and antagomir-Let-7i (Let-7i inhibitor) regenerated Pgrmc1 expression and lead in a significant elevation in mature BDNF level, which promotes neuronal differentiation, cellular survival, synaptic plasticity, and neurogenesis, despite precursor BDNF (pro-BDNF) levels did not change significantly, which is linked with neuronal apoptosis [1, 106]. Thus, the combination of progesterone and antagomir-Let-7i administration decreased the ischemic injury and improved the motor function, most likely by indirectly increasing mature BDNF levels. Another study analyzed the function of BDNF on miRNA profiling in human endothelial progenitor cells (EPC) as these cells show protective effect against cerebral vascular integrity in brain endothelium after ischemia [108, 109]. Results showed that BDNF can inversely modulate the expression of miR-3928 and positively modulate miR-636 and miR-485-5p. Importantly, BDNF treatment significantly inhibited miR-433 and induced miR-181c concentration, which might improve regenerative effect of EPCs in ischemia [108].

The first study in humans which analyzed the relation between BDNF and miRNAs in ischemic stroke was conducted by Zeng et al. The study first examined circulating miRNA profiles in the ischemic stroke patients. Further, *in silico* analysis showed that hypoxia-related miR-210 is a target of *BDNF* gene. The study documented the improvement of long-term outcomes for stroke therapy following the delivery of lentivirus-mediated miR-210 to the ischemic brains of mice. MiR-210 upregulated mature-BDNF/pro-BDNF ratio shows that miR-210 can be a promising therapeutic approach for ischemic stroke [110]. The latest human study results showed that BDNF serum levels were significantly lower in acute ischemic stroke patients compared to healthy individuals. Based on *in silico* prediction, miR-124 was identified responsible for regulating BDNF transcription which was further confirmed *in vitro* by using human neuronal cell lines. MiR-124 was higher in stroke patients and presented significant negative correlation between BDNF concentrations. Moreover, miR-124 levels correlated positively with stroke severity measured by National Institute of Health Stroke Scale (NIHSS) whereas BDNF correlated negatively with NIHSS score. This results indicates that (i) both miR-124 and BDNF can be a promising novel biomarker of ischemic stroke, (ii) miR-124 and BDNF could be a predictors of stroke severity, and (iii) inhibitor of miR-124 could be used as a potential approach for increasing BDNF serum levels [111]. Another *in silico* prediction showed that miR-191a-5p may target neuronal calcium sensor 1 (NCS-1) and BDNF. Results confirmed that the NCS-1 3′UTR is a binding side for miR-191a-5p; however, results failed to display BDNF and its binding side for miR-191a-5p [112].

Several analytical strategies have been used in studies on ischemic stroke including *in silico* analysis with validation of the results in experimental models, or miRNA profiling approach with *in silico* analysis and validation of the results by using *in vitro*/*in vivo* analysis. Based on bioinformatic tool and *in vitro*/*in vivo* experiments, Let-7i and miR-124 were the most promising miRNA biomarkers for stroke severity. Importantly, miR-210 was identified as an important biomarker of ischemic stroke both in microarray analysis by using human blood as well as by using bioinformatic tool. Essentially, its positive effect was confirmed in animal model. Ultimately, further studies should focus on confirming the importance of these three promising miRNAs, i.e., Let-7i, miR-124, and miR-210 in ischemic stroke (Fig. 3, Table 1).

## Is Targeted Delivery a Cure for Neurodegenerative and Neurovascular Diseases?

**Fig. 2:**
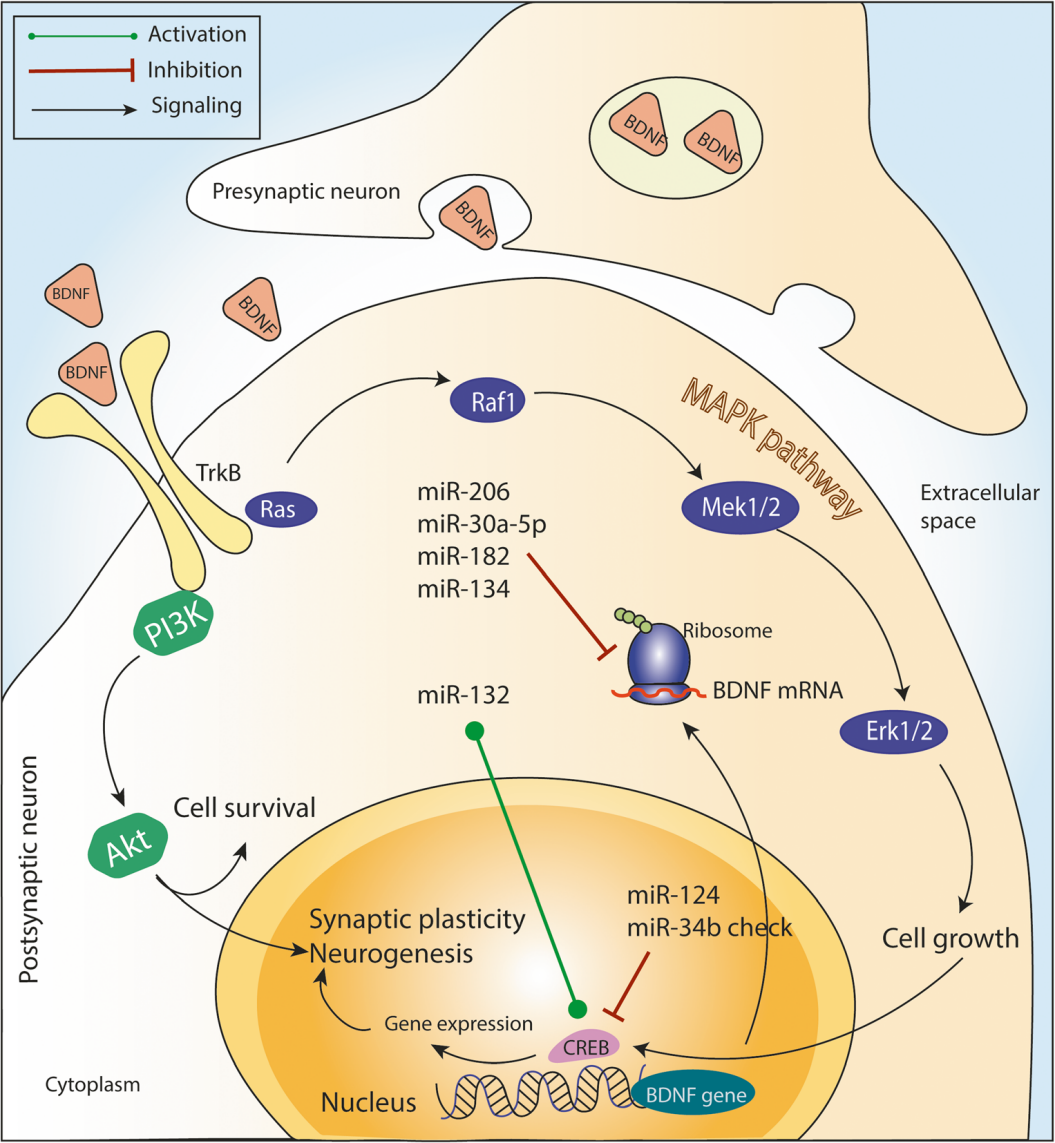
General illustration. Binding of BDNF to TrkB receptors initiates the signaling of several different pathways. The MAPK pathway induces cell growth and *CREB* and *BDNF* gene expression within the nerve nuclei. Additionally, *BDNF* expression induces BDNF protein synthesis in ribosomes. It is here that several miRNAs (miR-206, miR-30a-5p, miR-182, miR-134) can inhibit BDNF mRNA expression. This is the primary reason why studies have focused on using miRNA inhibitors to repair the neurodegenerative effect. Additionally, miR-132 can activate *CREB* and increase *BDNF* expression. Thus, this protein may improve synaptic plasticity and neurogenesis. Abbreviation: MAPK, mitogen-activated protein kinase; BDNF, brain-derived neurotrophic factor; CREB, cAMP response element-binding protein; miR, microRNA; TrkB, tyrosine kinase receptor B; PI3K, phosphoinositide 3-kinase; AKT, protein kinase B; ERK, extracellular signal-regulated kinase; Mek, MAP kinase

**Fig. 3:**
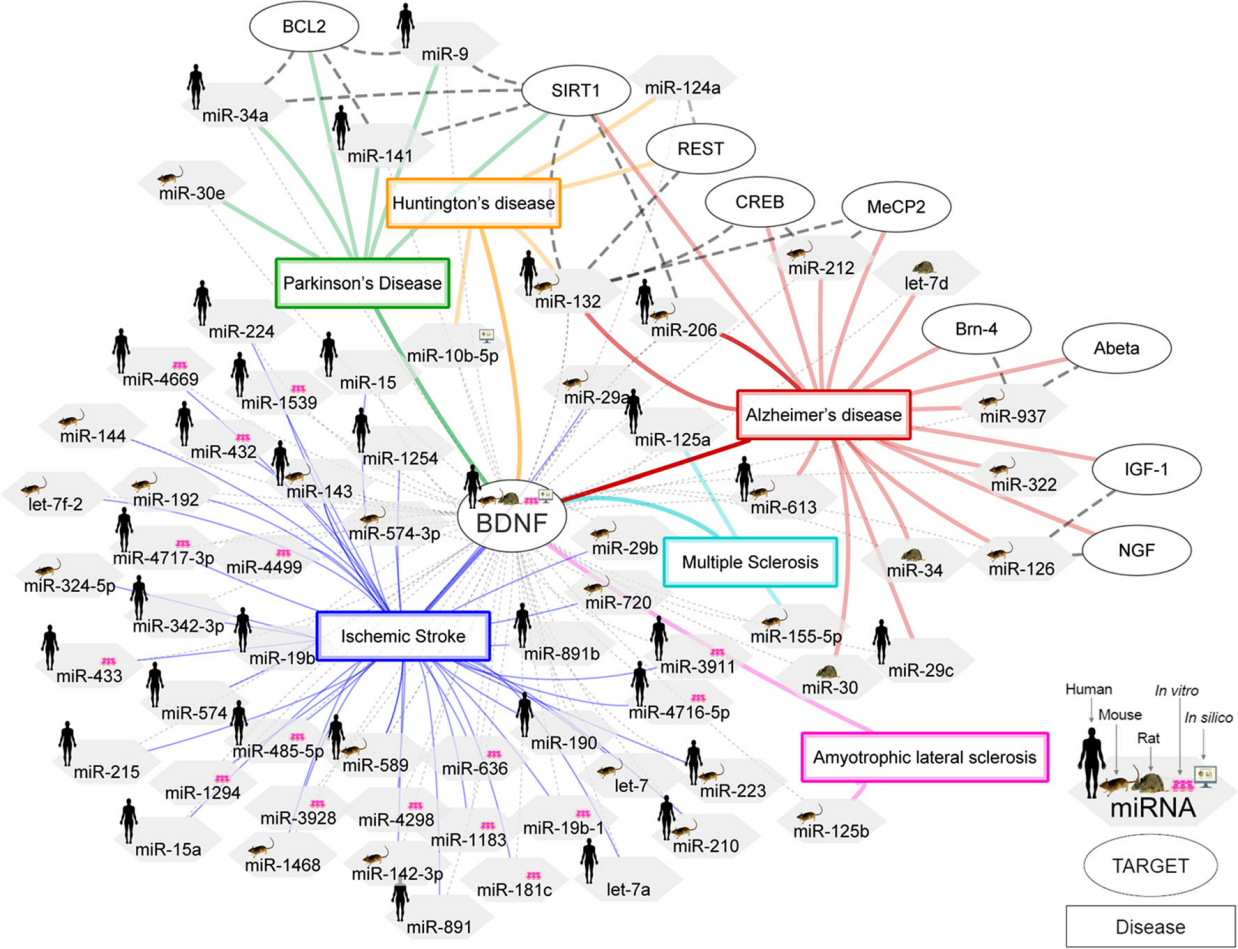
Network of BDNF signaling regulation by targeting miRNAs in neurodegenerative diseases and ischemic stroke in human/animal/*in vitro*/*in silico* studies. Abbreviations: BCL2, B-cell lymphoma 2; SIRT1, Sirtuin 1; REST, RE1-silencing transcription factor; CREB, cAMP response element-binding protein; MeCP2, methyl CpG binding protein 2; Abeta, Amyloid beta peptide- Aβ; IGF-1, insulin-like growth factor 1; NGF, nerve growth factor; BDNF, brain-derived neurotrophic factor; miRNA-miR, microRNA

Among the various BDNF–miRNA interactions discussed above, some miRNAs expressed in neurological tissues or cells are able to up-/downregulate BDNF expression. MiR-206, being one of the most well-studied miRNAs in AD, has been described to be implicated in the pathogenesis of AD due to suppression of BDNF expression in the brain. Previous studies have found that both in animal models and human studies of AD, the level of miR-206 is increased in the brain tissue and may contribute to memory impairment by blocking BDNF expression [38, 40, 42]. Consequently, some animal studies have used antagomiR-206 (inhibitor of miR-206) as a treatment and it further ameliorated memory impairments and BDNF dysfunction. Published data have suggested that inhibitor of miR-206 can be a novel therapeutic approach for AD. Similarly, miR-30e has been studied intensively in PD. The miR-30e agomir significantly improved motor function and inhibited dopamine neurons depletion, specifically by targeting and decreasing the inflammasome activity of the protein coding gene, *NLRP3.* Further, the gene increased inflammatory cytokines, such as COX-2, TNF-α, and iNOS. Importantly, the miR-30e agomir treatment restored the reduced production of BDNF in SNpc. Ultimately, this may suggest that miR-30e can have a protective function against neurodegeneration by targeting BDNF in PD [55]. However, these promising miRNAs targeted with BDNF have not been studied with other neurodegenerative diseases. Thus, further researchers should consider using these miRNA-based strategies in HD, ALS, MS, and ischemic stroke therapy. Currently, an effective cure for neurodegenerative diseases does not exist and, based on above described observations, an ideal solution to this issue at hand would be to develop targeted drug delivery of promising protective miRNAs or utilize miRNA inhibitors that are capable of inducing BDNF. New transport techniques such as nanospheres, nanovesicles, nanoparticles, and nanoexosomes could be a possible vehicle for the delivery of ago/antagomiRs in neurodegenerative tissues, which could then essentially regulate BDNF activity (Figs. 2 and 3).

## Concluding Remarks and Limitations

One of the advantages offered by the use of both circulating and extracellular miRNAs, as disease biomarkers, is their ability to distinguish between different etiologies of many different types of cardiovascular diseases and cancers, including brain tumors as well as neurological disorders [113–118]. Moreover, studies showed that BDNF plays a crucial role in neurodegenerative disorders. Apart from neurodegenerative disorders, the importance of BDNF in many other diseases such as diabetes and cancer was also investigated [1, 119]. However, limited studies showed the BDNF–miRNA interactions and the modulation of BDNF signaling by miRNAs. Protein–RNA interaction-analyzing tools and techniques are useful in understanding how BDNF and miRNAs behave following neurodegeneration and neuroregeneration, as well as in learning, memory, and cognition. Understanding the role of BDNF–miRNA interactions and how they modulate regrowth or repair of the nervous tissue in the CNS and peripheral nervous system will be useful. Numerous studies have shown that several miRNAs could pose as promising diagnostic and prognostic biomarkers in neurodegenerative and neurovascular diseases. Limited studies provide promising miRNA-based novel treatments. The limitation of the collective literature so far is how exactly BDNF and miRNAs interact on a molecular level. Besides, many miRNAs are still yet to be studied. A myriad of questions remain, including (i) Do this interaction mutually activate/inactivate each other’s transcription? (ii) Which signaling pathway could be activated by BDNF-targeted miRNAs? and (iii) Do they bring about any epigenetic alterations in their respective miR-binding sites? This is the reason further analyses are needed to explain these questions. Moreover, computational approaches are an important and cost-effective step in understanding the regulatory role of miRNA and the identification of their targets. Due to a large number of targets of each miRNA, it is unrealistic to rely only on wet-lab biological experiments. Therefore, currently suggested is integrating *in silico* target predictions with the biological knowledge including expression datasets [120]. This approach can be also useful in discovery of novel biomarkers and therapeutics. Using computational tools and tissue-specific expression profiles can help in identification of key regulators of the disease. *In silico* analysis should be performed to predict the novel miRNAs targeted to BDNF for neurodegenerative and neurovascular diseases; further experimental analysis should be performed for the validation of the prediction analysis.

## Abbreviations

BDNF: Brain-derived neurotrophic factor
CNS: Central nervous system
TrkB: Tropomyosin receptor kinase B
AD: Alzheimer’s disease
PD: Parkinson’s disease
HD: Huntington’s disease
ALS: Amyotrophic lateral sclerosis
MS: Multiple sclerosis
MiRNAs: MicroRNAs
CSF: Cerebrospinal fluid
NFG: Nerve growth factor
NT: Neurotrophins
NPY: Neuropeptide Y
KO: Knockout
CREB: cAMP response element-binding protein
Aβ1–42: Amyloid-beta 1–42 peptides
MSCs: Mesenchymal stem cells
As-miR-937: Antisense miRNA-937
Aβ: Amyloid-beta
DAT: Dementia of Alzheimer’s disease type
MCI: Mild cognitive impairment
DNMT3: DNA methyltransferase 3
WT: Wild type
aMCI: Amnestic mild cognitive impairment
LRRK2: Leucine-rich repeat kinase 2
MPP^+^: 1-Methyl-4-phenylpyridinium
ROS: Reactive oxygen species
PPARα: Peroxisome proliferator-activated receptor alpha
PPAR: Peroxisome proliferator-activated receptor
DHA: Docosahexaenoic acid
SNpc: Substantia nigra pars compacta
NLRP3: NLR family pyrin domain containing 3
POMC: Proopiomelanocortin
PVN: Paraventricular nucleus
VMN: Ventromedial nucleus of the hypothalamus
α-MSH: Alpha-melanocyte-stimulating hormone
MC4R: Melanocortin-4 receptor
Htt: Huntingtin
REST: RE1 silencing transcription factor
BzATP: 2′-3′-*O*-(benzoyl-benzoyl) ATP
Lin-: Lineage-negative
EAE: Experimental autoimmune encephalomyelitis
LIF: Leukemia inhibitory factor
NAWM: Normal appearing white matter
Pgrmc1: Progesterone receptor membrane component 1
pro-BDNF: Precursor BDNF
EPC: Endothelial progenitor cells
NCS-1: Neuronal calcium sensor 1
